# Hsa_circ_0076931 suppresses malignant biological properties, down-regulates miR-6760-3p through direct binding, and up-regulates CCBE1 in glioma

**DOI:** 10.1042/BSR20211895

**Published:** 2022-01-06

**Authors:** Yanbin Ke, Shixing Su, Chuanzhi Duan, Yezhong Wang, Guobin Cao, Zelu Fang, Yonghua Tuo, Wei Li, Zhaotao Wang, Shizhen Zhang

**Affiliations:** 1 Neurosurgery Center, Department of Cerebrovascular Surgery, The National Key Clinical Specialty, The Engineering Technology Research Center of Education Ministry of China on Diagnosis and Treatment of Cerebrovascular Disease, Guangdong Provincial Key Laboratory on Brain Function Repair and Regeneration, The Neurosurgery Institute of Guangdong Province, Zhujiang Hospital, Southern Medical University, Guangzhou 510282, Guangdong, China; 2 Department of Neurosurgery, The Second Affiliated Hospital of Guangzhou Medical University, Guangzhou 510260, Guangdong, China

**Keywords:** CCBE1, glioma, hsa_circ_0076931, miR-6760-3p

## Abstract

The function of circular RNAs (circRNAs) in gliomas is as yet unknown. The present study explored role of hsa_circ_0076931 in glioma. circRNA expression profiles were identified via RNA-seq followed by qRT-PCR validation in three pairs of glioma and normal brain tissues (NBT). The function of hsa_circ_0076931 was investigated *in vitro* using cell lines as well as *in vivo* using a xenograft tumor. Hsa_circ_0076931 was up-regulated by overexpression and an mRNA profile compared with wild-type was identified by RNA-seq. The relationship between miR-6760-3p and hsa_circ_0076931 or CCBE1 was confirmed via luciferase reporter or AGO2-RIP assays. A total of 507 circRNAs were identified in glioma tissues that were differentially expressed compared with that in NBT, and the sequencing data were deposited in BioProject (ID: PRJNA746438). Hsa_circ_0007694 and hsa_circ_0008016 were memorably increased whereas hsa_circ_0076931 and hsa_circ_0076948 decreased in glioma compared with those in NBT. Additionally, hsa_circ_0076931 expression was negatively correlated with histological grade. Overexpression of hsa_circ_0076931 inhibited proliferation, migration, and invasion while promoting apoptosis of glioma cells. A total of 4383 and 537 aberrantly expressed genes were identified between the hsa_circ_0076931-overexpressed and control groups in H4 and U118-MG cells, respectively; the sequencing data were deposited in BioProject (ID: PRJNA746438). These differentially expressed genes were mainly enriched in cancer-related pathways. In addition, elevated hsa_circ_0076931 levels induced the expression of CCBE1 while suppressing miR-6760-3p expression. miR-6760-3p can bind to hsa_circ_0076931. The experimental evidence supports using hsa_circ_0076931 as a marker for glioma and to help prevent malignant progression. The mechanism might be relevant to miR-6760-3p and CCBE1.

## Introduction

Gliomas are tumors in the central nervous system that originate from glial or neural stem cells and are the most common primary central nervous system tumor, representing approximately half of all primary intracranial tumors [1,2]. Unfortunately, the morbidity and mortality rates associated with glioma have rapidly increased recently, especially in developing countries [1]. Although improvements in glioma diagnosis and therapeutic strategies have been achieved, the therapeutic efficacy remains poor, with a median overall survival of <16 months [2]. Therefore, it is urgent and vital to identify the underlying molecular mechanisms of glioma initiation and progression to find potential strategies for glioma therapy.

Circular RNAs (circRNAs) are a kind of endogenous RNA that lack free 3′ and 5′ ends and can be stably present in cells and tissues [3]. Multiple lines of evidence highlight that circRNAs act as biomarkers for cancer diagnosis and as potential therapeutic targets [4–7]. Accumulating evidence indicates that circRNAs play a vital role in the development and progress of glioma. Hsa_circ_0006404, encoded by human *FOXO3*, is a promising biomarker for glioma diagnosis and prognosis [8]. Overexpressed circular E-cadherin RNA, which encodes the oncogenic E-cadherin variant, promotes glioma stem cell tumorigenicity by activating the EGFR-STAT3 signaling pathway [9]. Additionally, the amount of circ-FBXW7 is positively associated with overall survival of patients with glioblastomas [10]. Although circRNAs are known to play vital roles in glioma progression, the circRNA expression profile of gliomas and their detailed mechanisms remain unknown.

CircRNAs are thought to act as competitive endogenous RNAs (ceRNAs) that sequester target microRNAs (miRNAs) and diminish the repressive effects on downstream miRNAs molecules [11]. Some evidence suggests that the ceRNA network plays a specific role in glioma progression. For instance, the up-regulation of circATP5B contributes to glioma stem cell proliferation by acting as a miR-185-5p sponge [12]. In addition, the up-regulation of circ-ASPH has been shown to promote the proliferation and aggressiveness of glioma cells by modulating miR-599/AR/SOCS2-AS1 [13]. Hsa_circ_0110757 acts as an hsa-miR-1298-5p sponge to up-regulate ITGA1 and facilitate temozolomide resistance in gliomas [14]. However, the potential pathogenic role of the regulatory network among circRNA/miRNA/mRNAs has not been fully explored in glioma progression.

To identify the circRNA profile in gliomas, the present study conducted high-throughput sequencing on glioma samples [15]. We first investigated the underlying roles of a novel circRNA, hsa_circ_0076931, encoded by BAI3, in glioma progression. Furthermore, we explored the effects of the miR-6760-3p/CCBE1 axis on glioma cells. The results of the present study will expand our knowledge of the potential pathogenesis of the circRNA/miRNA/mRNA regulatory network and provide some potential therapeutic options for glioma.

## Methods

### Patients

A total of 41 glioma samples and 37 normal brain tissues (NBT) were obtained for the present study. The study was approved by the Institutional Review Board of the Second Affiliated Hospital of Guangzhou Medical University (approval number: 2020-hs-44), and written informed consent was obtained from the guardians of all subjects.

### CircRNA sequencing analysis

Total RNA was isolated from samples using TRIzol Reagent (Thermo Fisher, # 15596026). The concentration and quality of RNA were determined using an ND-2000 Spectrophotometer. For circRNA sequencing, linear RNA was removed from each sample using RnaseR. Subsequently, total RNA was digested using RNase R (Epicenter, Madison, WI, U.S.A.). Then, 1 μg RNA and the VAHTS mRNA-seq v2 Library Prep Kit for Illumina (Vazyme Biotech, China) were used for library preparation. Libraries were subjected to deep sequencing with an Illumina HiSeq 3000 at Guangzhou Forevergen Biotechnology Co., Ltd. circRNAs with |log2 Ratio| > 0.6 and *P*<0.05 were considered differentially expressed, and differently expressed circRNAs were selected to conduct heatmap and hierarchical clustering analyses. The sequencing data were deposited in BioProject (ID: PRJNA746438).

### Analyzing the expression of hsa_circ_0076931 using agarose gel electrophoresis

Divergent and convergent primers were run on a 1.5% agarose gel at 100 V for 20 min with 1× TAE (Tris Acetate EDTA) buffer.

The circling hsa_circ_0076931 bands were visualized by staining the gel with ethidium bromide. Images were captured using a gel documentation unit. The product of divergent primers was sent for sequencing to Sangon Biotech Co., Ltd. (Shanghai, China).

### Quantitative real-time polymerase chain reaction

Total RNA was isolated from glioma samples, NBT, and glioma cells (U118-MG, H4, SK-N-MC, and U251 cells) using Column Animal RNAOUT (TIANDZ, #3070). RNA concentration was determined using an ND-2000 Spectrophotometer (Thermo Fisher Scientific), and quantitative real-time polymerase chain reaction (qRT-PCR) was performed using the KAPA SYBR FAST qPCR Kit (Kapa Biosystems) and a 7300 Real-Time PCR System (Applied Biosystems). mRNA primer pairs are listed in Table 1. The 2^−ΔΔCT^ method was used for qRT-PCR data analysis [16].

**Table 1:** Information of primers

Gene name	Sequence (5′-3′)
hsa_circ_0007694-divergent primer-F	ATGGAAATCTAATGGAGGTGGAGG
hsa_circ_0007694-divergent primer-R	GCTTTTTGATGAAGGATGCCT
hsa_circ_0008691-divergent primer-F	TAGAACGGAAGACCAGCTCC
hsa_circ_0008691-divergent primer-R	GCTTTAAGTGCCTTGTGACCG
hsa_circ_0076931-divergent primer-F	GATAACAGGGCAGCAATGTGAAG
hsa_circ_0076931-divergent primer-R	ACTCTTCCACACCAGATTCACCA
hsa_circ_0076931-convergent primer-F	CGGCCCATTAAGAGAATCAA
hsa_circ_0076931-convergent primer-R	CTCTTGCCACTGTCCATCAA
hsa_circ_0076948-divergent primer-F	GTCACAATAAGGCCTGAACCC
hsa_circ_0076948-divergent primer-R	TTGAAAGAAGTTGTTTTGGAGTCA
CCBE1-F	TCGCGACGACTAAATACCCG
CCBE1-R	TCGCGACGACTAAATACCCG
GADPH-F	GAGTCAACGGATTTGGTCGT
GADPH-R	GACAAGCTTCCCGTTCTCAG
hsa-miR-6760-3p-RT	GTCGTATCCAGTGCAGGGTCCGAGGTATTCGCACTGGATACGACCTGGGG
hsa-miR-6760-3p-F	ATTATCACACTGTCCCCTTCTC
Universe-R	GTGCAGGGTCCGAGGT
hsa-U6-F	CTCGCTTCGGCAGCACA
hsa-U6-R	AACGCTTCACGAATTTGCGT

### Cell culture

The H4 and SK-N-MC cell lines were purchased from the American Type Culture Collection. U118-MG and U251 cell lines were purchased from the National Collection of Authenticated Cell Cultures. SK-N-MC cells were expanded in minimum essential medium (Gibco, #41500-034) containing 10% fetal bovine serum (FBS; Gibco, #16000-044). U251 cell lines were grown in streptomycin (Gibco, #15140122) at 37°C with 10% FBS. In addition, U118-MG, H4, and U251 cell lines were maintained in Dulbecco’s phosphate-buffered saline with 10% FBS (Gibco, #16000-044).

### Overexpression of hsa_circ_0076931 in U118-MG and H4 cells

To overexpress hsa_circ_0076931 in U118-MG and H4 cells, the front wing of the cyclization sequence of hsa_circ_0076931 (5′-AGTGCTGAGATTACAGGCGTGAGCCACCACCCCCGGCCCACTTTTTGTAAAGGTACGTACTAATGACTTTTTTTTTATACTTCAG-3′) and the posterior wing of the cyclization site (GTAAGAAGC AAGGAAAAGAATTAGGCTCGGCACGGTAGCTCACACCTGTAATCCCAGCA) were cloned into the LV003 vector (GENERAL BIOL, Anhui, China). When U118-MG and H4 cells were cultured to approximately 80% confluence, empty vector (LV003) and LV003-hsa_circ_0076931 were transfected into the cells using Lipofectamine 3000 reagent and cultured for 48 h. Transfection efficiency was determined via qRT-PCR.

### Cell viability assays

Cell viability was evaluated using the MTS test. The treated U118-MG and H4 cells were seeded in 96-well plates at a concentration of 5 × 10^3^ cells/well in a 200 μl of complete medium. Then 20 μl MTS liquid (#ab197010) was added to each well at 24, 48, and 72 h. After incubation for 4 h, the supernatant was discarded and 150 μl DMSO was added to each well and the samples were subjected to low-speed oscillation on a shaker for 10 min after. Absorbance values were measured at 490 nm using an automated microplate reader (Shanghai Flash Spectrum Biotechnology Co., Ltd., Shanghai, China). The growth curve was plotted with time as the *X*-axis and OD as the *Y*-axis.

### Apoptosis analysis

The Annexin V-FITC/PI kit (Biolegend, California, U.S.A.) was used to detect cell apoptosis as per the manufacturer’s instructions. After transfection, U118-MG and H4 cells growing at the logarithmic growth phase were harvested and digested with 0.25% trypsin for 2 min. The cells were centrifuged and then washed twice with cold PBS. The cells were resuspended in 500 μl of binding buffer to a final concentration of 10^6^ cells/ml. Next, 100 μl of the cell suspension was transferred to a 5-ml flow tube into which 5 μl FITC and 5 μl PI were added. The contents of the flow tube were mixed and incubated for 15 min in the dark. A flow cytometer (Beckman, Florida, U.S.A) was used to examine the cells, and FlowJo 7.0.1 software (Tree Star, Ashland, Oregon) was used to analyze the data.

### Cell migration assays

Cell migration assays were used to determine the motility of U118-MG and H4 cells. First, 1 × 10^6^/ml cells were resuspended in 100 μl of serum-free medium and seeded in the inner chamber. Next, the bottom chamber was incubated with three replicates of 600 μl medium containing 20% FBS for 24 h at 37°C in a humidified atmosphere of 5% CO_2_. The cells on the basolateral chamber were fixed with 4% paraformaldehyde for 10 min, stained with 1% Crystal Violet for another 10 min, washed with cold PBS once, and then photographed using a light microscope (OPTEC CCD TP510).

### Cell invasion assays

Matrigel (BD 356234) was dissolved overnight at 4°C. Next, 40 μl precooled DMEM supplemented with 1/7 matrigel was added to the precooled Transwell chamber. Then, the matrigel-covered Transwell filters were incubated at 37°C for 2 h to solidify the matrigel. Next, 100 and 600 μl DMEM were added to the top and bottom chamber, respectively. The DMEM was dropped after 24 h. Then, 1 × 10^6^/ml cells were resuspended in 100 μl serum-free medium and seeded in the inner chamber. The bottom chamber was incubated with 600 μl DMEM supplemented with 20% FBS for 24 or 48 h. The cells on the basolateral chamber were fixed with 4% paraformaldehyde for 15 min, washed with PBS, and stained with 1% Crystal Violet for 10 min, and then rewashed with cold PBS. A microscope was used to observe the cells that passed the small hole to the bottom chamber, take pictures, and count the number of cells that passed.

### Construction of stable expression cell lines in U118-MG cells

Lentivirus (oe-hsa_corc_0076931 and oe-NC) was provided by Genepharma (Shanghai, China) and used to infect U118-MG cells according to the manufacturer’s protocol.

### Tumor formation in nude mice

Twelve BALB/c mice (6 weeks old) were purchased from the Guangdong Medical Experimental Animal Center. They were randomly divided into two groups: oe-NC and oe-hsa_corc_0076931. The oe-hsa_corc_0076931 U118-MG cells and oe-NC U118-MG cells were subcutaneously injected (5 × 10^6^ cells in 0.1 ml PBS) into the right lower flank of the mice. The tumor volume was tested for 3 d until the tumor length reached 8 mm. Thereafter, the mice were sacrificed using the CO_2_ method, and the tumor was collected for further study. Our animal experiment was conducted in Forevergen (Guangzhou, China) and was approved by the Forevergen Biosciences Experimental Animal Ethics Committee (approval number: IACUC-G16044).

### Immunohistochemistry

Immunohistochemistry (IHC) detected the expression of Ki67 in tumor tissue. The tumor tissue paraffin sections were deparaffinized, and endogenous peroxidase activity was blocked by incubation with 3% H_2_O_2_. The blocked sections were incubated with anti-Ki67 antibody (dilution 1:100; Boster, A00254) at 4°C overnight and then sequentially incubated with a biotin-labeled secondary antibody. The sections were then stained with 3,3′-diaminobenzidine. Finally, the sections were counterstained using hematoxylin and fixed. For each section, three fields of view were randomly selected and photographed under 200× magnification.

### mRNA sequencing after overexpression of hsa_circ_0076931 in H4 and U118-MG cells

To detect changes in mRNA expression profiles after the overexpression of hsa_circ_0076931 in H4 and U118-MG cells, we collected cells and extracted RNA for mRNA high-throughput sequencing. Briefly, polyA mRNA was purified via hybridization to Dynaloligo beads, the RNA was fragmented, and double-stranded complementary DNA (cDNA) was synthesized. End repair and A-addition were performed to ligate the cDNA fragments to adapters. The ligated cDNA was subjected to PCR amplification, and library quality was assessed using an Agilent Bioanalyzer 2100 (Agilent Technologies). RNA sequencing (RNA-seq) was performed using the Illumina Hiseq 3000 (Illumina, San Diego, CA, U.S.A.) at Guangzhou Forevergen Biotechnology Co., Ltd. R software was used for quantile normalization and subsequent data processing. Differentially expressed mRNAs were screened according to fold change (FC) and *P* (*P*<0.05 and FC > 1.5 or *P*<0.05 and FC < 0.67). Gene Ontology (GO) and Kyoto Encyclopedia of Genes and Genomes (KEGG) pathway enrichment analyses were performed to identify potential biological processes of the differentially expressed mRNAs based on the GO (http://geneontology.org/page/go-enrichment-analyses) and KEGG pathways (http://www.genome.jp/kegg/pathway.html) databases. The *P-*value of each GO term was calculated from right-sided hypergeometric tests. Benjamini-Hochberg adjustment was used for multiple test correction [17,18]. Terms with a *P*-value < 0.05 were considered notably enriched. The KEGG pathway enrichment analysis was conducted by DAVID. All sequencing data were deposited in BioProject (ID: PRJNA746438).

### Construction of circRNAs-miRNAs-mRNAs network

First, we chose altered mRNAs, which mediated by hsa_circ_0076931 in U118-MG and H4 cells. Then, miRNAs targeting the above mRNAs were predicted by TargetScan (release v.7.1) and miRanda (v.3.3a) [19,20]. Finally, based on the interaction relationship among hsa_circ_0076931-miRNAs-mRNAs, ceRNA networks were constructed and visualized by Cytoscape software.

### Western blotting

Treated cells were washed thrice with cold PBS, and proteins were extracted with the RIPA Lysis and Extraction Buffer (Med Chem Express, no. HY-K1001). Protein concentration was quantified using the BCA Protein Assay Kit (Thermo Fisher, no. 23225). Then, Western blotting was performed as previously described [21]. The following antibodies were used: anti-CCBE1 (NBP1-79501, dilution 1:1000, NOVUS) and anti-GAPDH (60004-1-Ig, dilution 1:8000, Proteintech).

### Luciferase reporter assay

Dual fluorescein reporter gene detection was conducted using a Dual-Luciferase Assay System Kit (Promega, Madison, WI, U.S.A.) according to the manufacturer’s instructions. Bioinformatic analysis predicts that hsa_circ_0076931 and miR-6760-3p have two potential binding sites at 432-452 bp and 504-524 bp, respectively [22]. Wild-type (WT) (432-452 bp), WT (504-524 bp), mutant (MUT) (432-452 bp), and MUT (504-524 bp) of hsa_circ_0076931 3′ UTRs were amplified and cloned into the luciferase reporter vector pmirGLO (Promega). HEK293T cells were co-transfected with luciferase plasmids and miR-6760-3p mimics. After 24 h, luciferase activity was measured using the Dual-Luciferase Reporter Assay System (Promega), and firefly luciferase activity was normalized against Renilla luciferase activity.

### AGO2-RIP assay

The binding of hsa_circ_0076931, miR-6760-3, or CCBE1 to AGO2 proteins was examined using the RIP kit (Millipore, U.S.A.). U118-MG cells were cleaned, collected, and resuspended with an equal volume of RIPA lysate (Beyotime, China, P0013B). After centrifugation, some samples were removed as input and some were incubated with antibodies for co-precipitation. Magnetic beads (50 μl) were suspended in RIP wash buffer (100 μl) from each co-precipitation reaction system and combined with AGO2 (5 μg, AB32381, 1∶50, Abcam, U.K.), and IgG (5 μg, 1∶100, AB109489, Abcam, U.K.). The magnetic bead-antibody complex was suspended in 900 μl RIP wash buffer and then incubated with 100 μl cell extracts at 4°C overnight. Then, the complex was collected and digested using proteinase K. Finally, the levels of hsa_circ_0076931, miR-6760-3, and CCBE1 were tested by qRT-PCR.

### Statistical analyses

All experiments were repeated at least thrice *in vitro*, and all data were analyzed using GraphPad Prism v.7. Also, ImageJ software was used to perform cell-count statistics on cell migration and invasion results and protein grayscale analysis on western blot. The data are presented as mean values ± standard deviation (SD). Differences were analyzed for significance (*P*<0.05) by one-way or two-way ANOVA using SPASS 18.0 (SPASS, Chicago, IL), which was followed by the Tukey’s post-hoc test.

## Results

### Analysis of circRNA expression profiles

**Figure 1:**
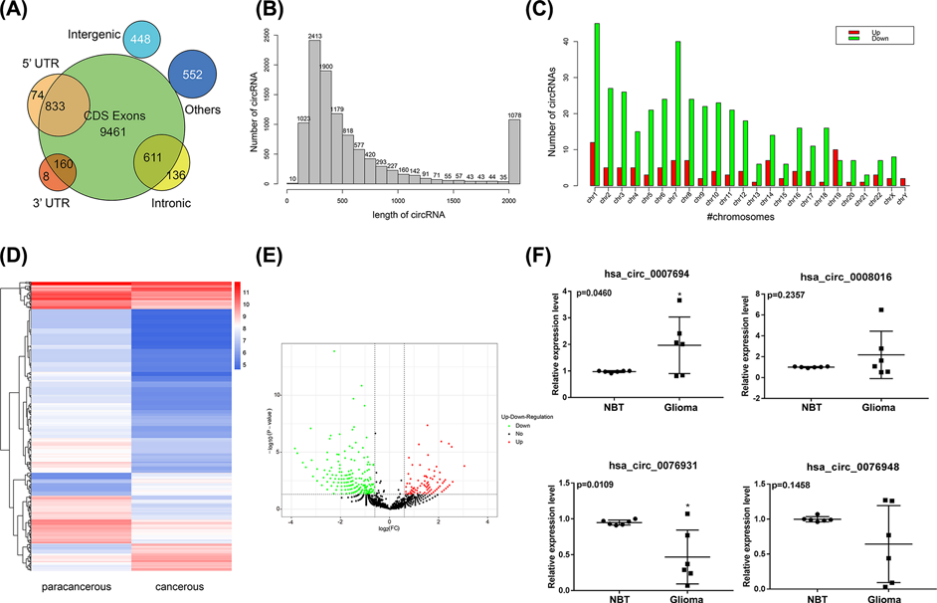
Microarray profile data of glioma versus normal brain tissues (NBT) (*n*=3) (**A**) Distribution of sources of circRNAs identified between glioma and NBT. (**B**) Distribution of length of altered circRNAs between glioma and NBT. (**C**) Chromosome distribution showing the numbers of up- and down-regulated circRNAs located in different chromosomes. (**D**) Hierarchical clustering showing a distinguishable expression pattern of circRNAs between glioma and NBT. (**E**) Volcanic maps showing a distinguishable expression pattern of circRNAs between glioma and NBT. (**F**) The level of hsa_circ_0007694, hsa_circ_0008016, hsa_circ_0076931, and hsa_circ_0076948 in glioma and NBT was examined by qRT-PCR (*n*=6, *P<0.05).

A total of 10,679 circRNAs were detected in high-throughput RNA-sequence analysis. About 88.59% (9461) of circRNAs were from protein-coding exons, 6.99% (747) were from intronic regions, 8.49% (907) were from 5′ UTRs, 1.57% (168) were from 3′ UTRs, and 4.20% (448) were from intergenic regions (Figure 1A). A total of 507 circRNAs exhibited a significant expression change (|Fold change| ≥ 0.6, *P*<0.05) between glioma and NBT, with 100 circRNAs up-regulated and 407 circRNAs down-regulated markedly. The identified circRNA transcripts were mainly 100–500 bp in length as confirmed by sequence length analysis (Figure 1B). In addition, we found that the different circRNAs were located at all chromosomes (Figure 1C). Up-regulated circRNAs were mainly located at chromosomes 1 and 19, whereas down-regulated circRNAs were located at chromosomes 1 and 7. A heat map and a volcano plot were used to show the differential circRNA expression profiles between glioma and NBT (Figure 1D,E). The top 20 dysregulated circRNAs are listed in Table 2 (|Log2| ≥ 1.0, *P*<0.05). We confirmed the expression levels of hsa_circ_0007694, hsa_circ_0008016, hsa_circ_0076931, and hsa_circ_0076948 by qRT-PCR on the same samples used for the RNA-seq analysis. The qRT-PCR showed that the level of hsa_circ_0007694 and hsa_circ_0008016 was higher in glioma compared with NBT, whereas that of hsa_circ_0076931 and hsa_circ_0076948 was lower in glioma compared with NBT. Furthermore, hsa_circ_0007694 was up-regulated prominently, and hsa_circ_0076931 was down-regulated notably, in agreement with the RNA-seq analysis (Figure 1F).

**Table 2:** The top 20 differentially expressed circRNAs

circBase ID	Gene ID	Log2 Fold Change	Up-Down	*P*-value
hsa_circ_0007694	ATM	2.534	Up	3.39E-06
hsa_circ_0008016	FGFR1	2.418	Up	1.79E-06
hsa_circ_0002130	C3	2.108	Up	1.14E-06
hsa_circ_0071099	ARHGAP10	1.545	Up	4.32E-08
hsa_circ_0001519	MAN2A1	1.196	Up	2.06E-06
hsa_circ_0060424	PTPRT	−3.858	Down	4.71E-06
	LUZP2	−3.214	Down	8.14E-08
hsa_circ_0087357	UBQLN1	−2.436	Down	6.62E-07
hsa_circ_0017251	AKT3	−2.436	Down	6.62E-07
hsa_circ_0002980	AGK	−2.398	Down	3.44E-07
hsa_circ_0008587	RP11-37B2.1	−2.254	Down	1.36E-14
hsa_circ_0005918	FCHSD2	−2.029	Down	2.63E-07
hsa_circ_0072309	LIFR	−1.622	Down	2.98E-06
hsa_circ_0076931	BAI3	−1.52	Down	4.18E-06
hsa_circ_0076948	BAI3	−1.513	Down	1.05E-07
hsa_circ_0007715	CIRBP	−1.468	Down	2.02E-10
hsa_circ_0006156	FNDC3B	−1.445	Down	6.12E-08
	ATRNL1	−1.374	Down	1.44E-06
	KCNN2	−1.136	Down	1.45E-11
hsa_circ_0000994	SLC8A1	−1.085	Down	4.11E-06

### Hsa_circ_0076931 decreased in glioma tissue and was associated with clinical characteristics

**Figure 2:**
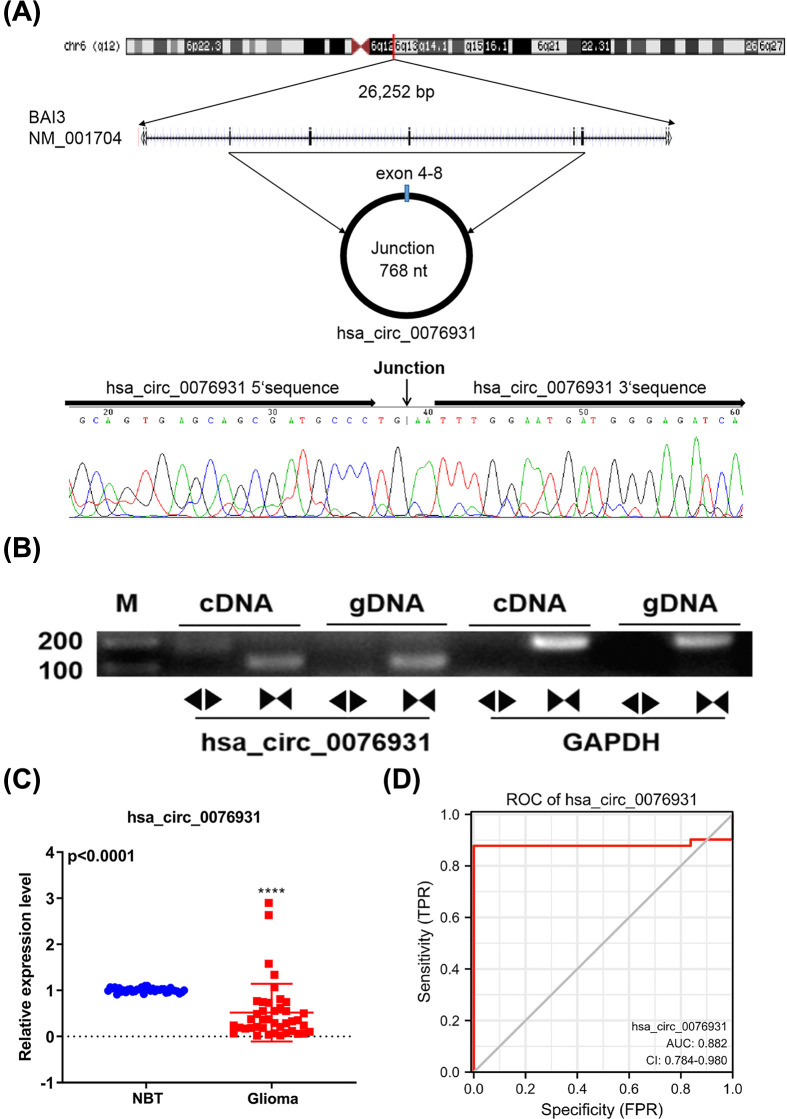
Significant decrease in the level of hsa_circ_0076931 in glioma tissues (**A**) Detection of the junction site in hsa_circ_0076931 via Sanger sequencing. (**B**) The cyclic nature of hsa_circ_0076931 was detected via agarose gel electrophoresis. (**C**) The level of hsa_circ_0076931 in glioma (*n*=41) and normal brain tissues (*n*=31) was examined using qRT-PCR (****P<0.0001). (**D**) The ROC curve analyses of hsa_circ_0076931 (AUC = 0.882) in glioma patients. AUC represents the area under the ROC curve.

Hsa_circ_0076931 is derived from regions of exons 4-8 of BAI3 (NM_001704), with a junction of 768 nt (Figure 2A). The junction cyclization site in hsa_circ_0076931 was analyzed by Sanger sequencing (Figure 2A). Next, specific convergent and divergent primers that specifically amplify the linear and back splicing forms of BAI3 were designed to confirm the presence of hsa_circ_0076931. We found that hsa_circ_0076931 could be amplified from cDNA but not genomic DNA (gDNA) templates (Figure 2B). hsa_circ_0076931 expression was detected in glioma tissues (*n*=41) and NBT (*n*=37) using qRT-PCR. The level of hsa_circ_0076931 was memorably decreased in glioma compared with NBT samples (*P*<0.0001, Figure 2C).

To explore whether the level of hsa_circ_0076931 correlated with clinical features of glioma and overall patient survival, we examined the expression of hsa_circ_0076931 in a 41-case cohort (Table 3). The level of hsa_circ_0076931 was markedly up-regulated in female glioma tissues (*n*=17, 0.7549 ± 0.193) compared with male glioma tissues (*n*=24, 0.3503 ± 0.084, *P*=0.0396). In addition, hsa_circ_0076931 expression was negatively correlated with pathological gradation (*P*=0.022). Glioma patients with high-grade, advanced pathological gradation (≥III) had lower expression of hsa_circ_0076931 (*n*=17, 0.2561 ± 0.042) compared with patients with low pathological graduation (<III, *n*=24, 0.7036 ± 0.154). However, hsa_circ_0076931 expression had no obvious correlation with age and overall survival (*P*=0.922 and *P*=0.290, respectively). The AUC of hsa_circ_0076931 was 0.882 in glioma patients (Figure 2D). The above results suggest that hsa_circ_007693 exerts an important role in glioma progression.

**Table 3:** Clinical sample information analysis

Groups	Number	Expression Of hsa_circ_0076931(Mean ± SEM)	*P* value
Ages			
≤45	22	0.5271 ± 0.1248	0.9222
>45	19	0.5076 ± 0.1572	
Gender			
Male	24	0.3503 ± 0.08392	0.0396
Female	17	0.7549 ± 0.193	
OS (overall survival)			
≤15 months	16	0.3873 ± 0.1735	0.2900
>15 months	25	0.6017 ± 0.1153	
Pathological gradation			
<III	24	0.7036 ± 0.1544	0.0220
≥III	17	0.2561 ± 0.04244	

### Overexpression of hsa_circ_0076931 inhibited proliferation, migration, and invasion while promoting apoptosis of glioma cells *in vitro*

**Figure 3:**
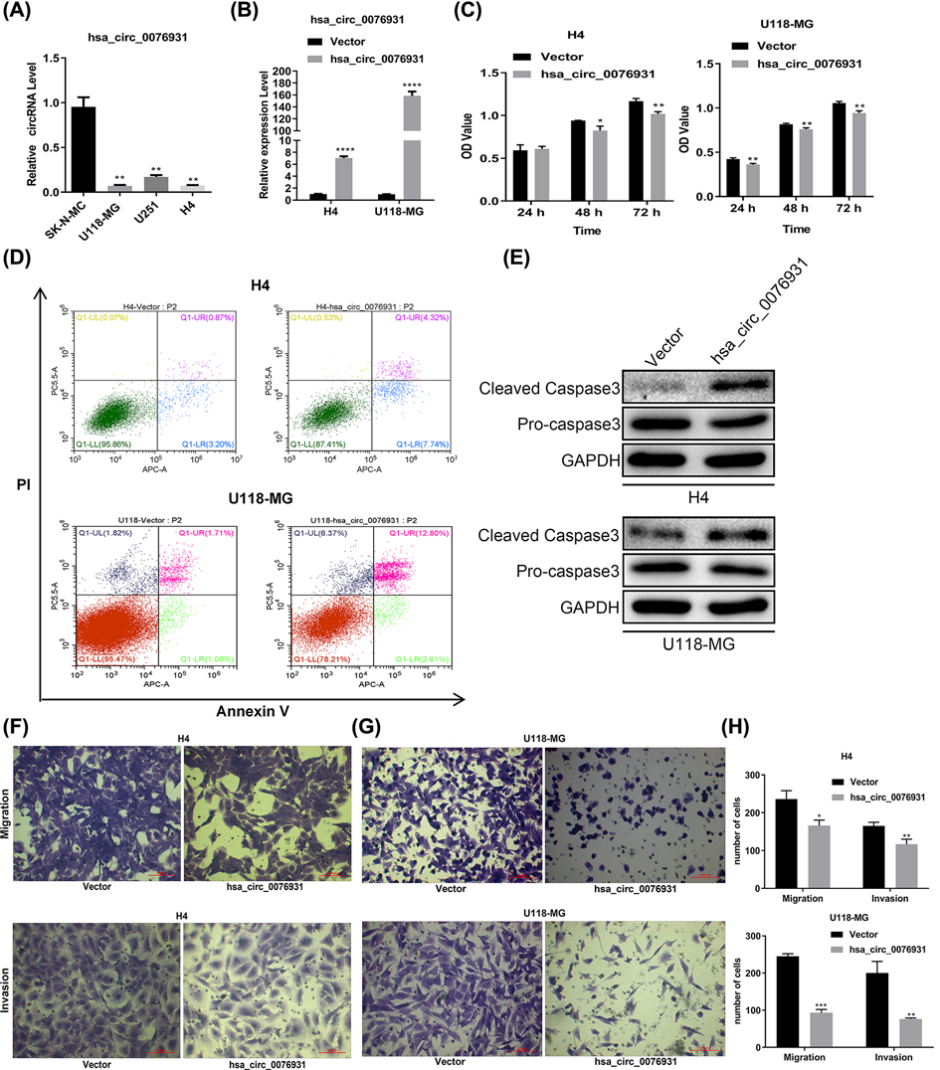
Overexpression of hsa_circ_0076931 inhibited proliferation, migration, and invasion of glioma cells *in vitro* (**A**) The level of hsa_circ_0076931 in U118-MG, H4, SK-N-MC, and U251 cells was examined by qRT-PCR. (**B**) The transfection efficiency of the hsa_circ_0076931 sequence in U118-MG and H4 cells was measured by qRT-PCR. (**C**) The cell viability of hsa_circ_0076931-elevated U118-MG and H4 cells was analyzed via MTS assay. (**D**) The apoptosis of hsa_circ_0076931-elevated U118-MG and H4 cells was analyzed using flow cytometry. (**E**) Pro-caspase3 and cleaved caspase3 expression were tested by immunoblotting in hsa_circ_0076931-overexpressed U118-MG and H4 cells. (**F–H**) The migration and invasion abilities of hsa_circ_0076931-elevated U118-MG and H4 cells were measured by Transwell assay. Data represent mean ± SD.; *n*=3, *P<0.05, **P<0.01, ***P<0.001, ****P<0.001.

We found that U118-MG and H4 cells expressed a low amount of hsa_circ_007693, whereas SK-N-MC and U251 cells expressed a high level of hsa_circ_007693 (Figure 3A). We overexpressed hsa_circ_007693 in U118-MG and H4 cells and confirmed overexpression by qRT-PCR (Figure 3B). The viability of U118-MG and H4 cells decreased in the hsa_circ_007693-overexpressed group, as confirmed by MTS assays (Figure 3C). In addition, the apoptosis rates of U118-MG and H4 cells increased in the hsa_circ_007693-overexpressed groups, as detected by flow cytometry analysis (Figure 3D). Furthermore, immunoblotting showed that hsa_circ_007693 overexpression could also up-regulate cleaved Caspase3 and down-regulate pro-Caspase3 in U118-MG and H4 cells (Figure 3E). Moreover, Transwell migration and invasion revealed fewer cells in the hsa_circ_007693-overexpressed group than in the control vector group (Figure 3F–H). The above data demonstrate that overexpression of hsa_circ_007693 can block the progression of glioma cells *in vitro*.

### Overexpression of hsa_circ_0076931 inhibited progression of glioma *in vivo*

**Figure 4:**
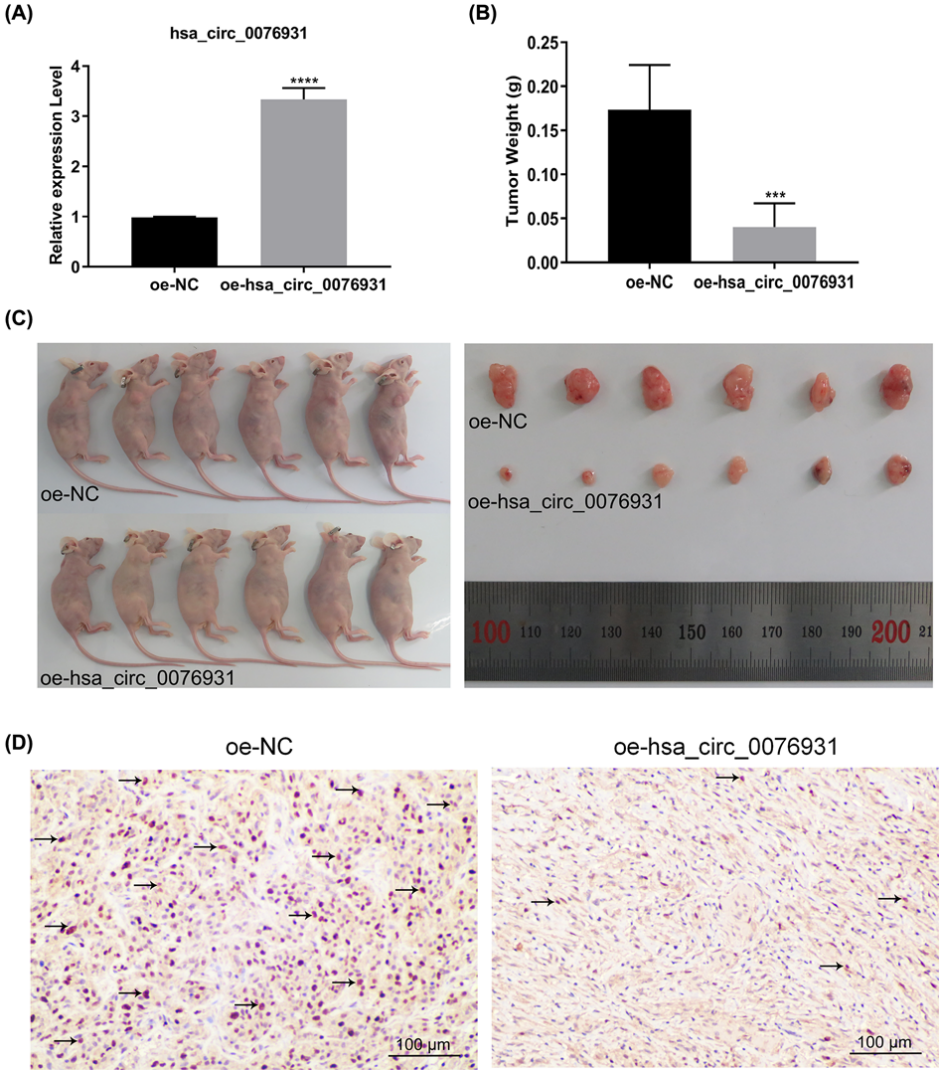
Overexpression of hsa_circ_0076931 blocked the growth of glioma cells *in vivo* (**A**) Stable overexpression of hsa_circ_0076931 in U118-MG cells was confirmed by qRT-PCR. (**B** and **C**) oe-hsa_circ_0076931 and oe-NC U118-MG cells were injected into BALB/C nude mice (*n*=6). Compared with the oe-NC group, tumor weight (B) and tumor size (C) were notably reduced in the oe-hsa_circ_0076931 group. (**D**) IHC showed that the expression of Ki67 prominently decreased in the oe-hsa_circ_0076931 group. Data represent mean ± SD.; *n*=3, ***P<0.001, ****P<0.001.

To evaluate the tumor-suppressing effects of hsa_circ_0076931 *in vivo*, we established U118-MG cells that stably overexpressed hsa_circ_0076931 (oe-hsa_circ_0076931) and corresponding control cells (oe-NC) and confirmed expression by qRT-PCR (Figure 4A). Compared with the oe-NC group, hsa_circ_0076931 overexpression dramatically reduced the mean tumor weight and tumor size (Figure 4B,C). In addition, decreased Ki67 expression was detected in the oe-hsa_circ_0076931 group compared with oe-NC (Figure 4D). Collectively, we showed that overexpressed hsa_circ_0076931 inhibited tumor growth *in vivo*.

### Identification of differentially expressed genes and signal pathways in cells overexpressing hsa_circ_0076931

**Figure 5:**
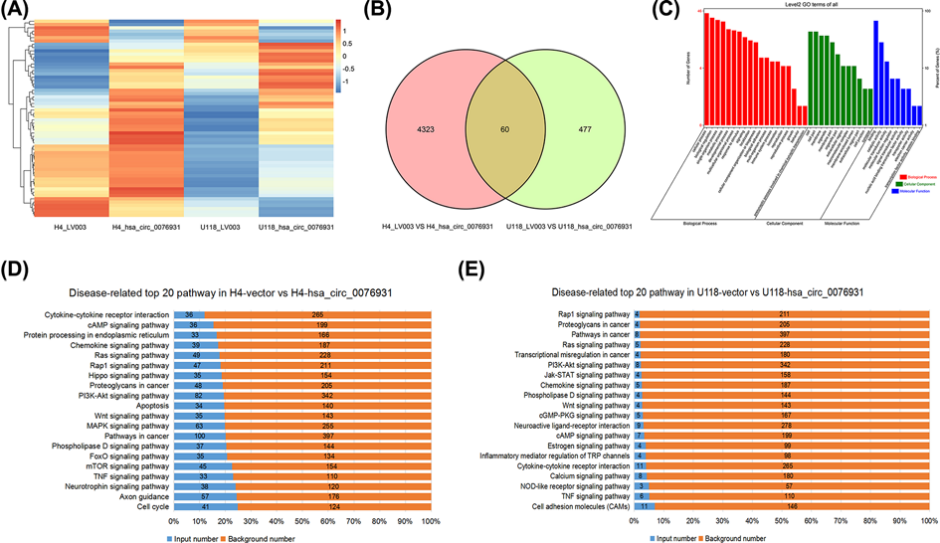
Profile of microarray data between wild-type H4/U118-MG and hsa_circ_0076931-elevated H4/U118-MG cells (**A**) Hierarchical clustering showed a distinguishable expression pattern of mRNAs between wild-type H4/U118-MG and hsa_circ_0076931-elevated H4/U118-MG cells. (**B**) The number of altered mRNAs in H4 and U118-MG cells induced by hsa_circ_0076931 up-regulation. (**C**) GO enrichment of differentially regulated mRNAs. (**D**) KEGG pathway enrichment analysis of differentially regulated mRNAs between H4 and hsa_circ_0076931-elevated H4 cells. (**E**) KEGG pathway enrichment analysis of differentially regulated mRNAs between U118-MG and hsa_circ_0076931-elevated U118-MG cells.

We used high-throughput sequencing analysis to investigate global changes to expression patterns in response to hsa_circ_0076931 up-regulation. Heat maps of the differentially expressed genes in the hsa_circ_007693-overexpressed U118-MG and H4 cells were generated using Cluster software (Figure 5A). Based on *P*-value < 0.05 and |FC| > 1.5, 4,383 and 537 aberrantly expressed genes were identified between the hsa_circ_0076931-overexpressed group and control group in H4 and U118-MG cells, respectively. Additionally, 60 genes were differentially expressed in both the H4 and U118-MG cells overexpressing hsa_circ_0076931 compared with the controls (Figure 5B). To further understand the role of hsa_circ_0076931 in glioma development, we analyzed the abnormally expressed genes for GO term enrichment and found that the principal biological processes included ‘cellular process,’ ‘biological regulation,’ ‘single-organism process,’ and ‘metabolic process.’ The top three cellular components included ‘cell part,’ ‘membrane,’ and ‘organelle.’ The primary molecular function was associated with ‘binding,’ ‘catalytic activity,’ and ‘molecular transducer activity’ (Figure 5C).

In the KEGG analysis, we found many essential pathways related to cancer development. The differentially expressed genes identified from the hsa_circ_0076931-up-regulated H4 and U118-MG cells were mainly enriched in the TNF signaling pathway, phospholipase D signaling pathway, pathways in cancer, Wnt signaling pathway, PI3K-Akt signaling pathway, proteoglycans in cancer, Rap1 signaling pathway, Ras signaling pathway, chemokine signaling pathway, the cAMP signaling pathway, and cytokine–cytokine receptor interaction (Figure 5D,E).

### Hsa_circ_0076931 stimulated CCBE1 expression by sponging miR-6760-3p

**Figure 6:**
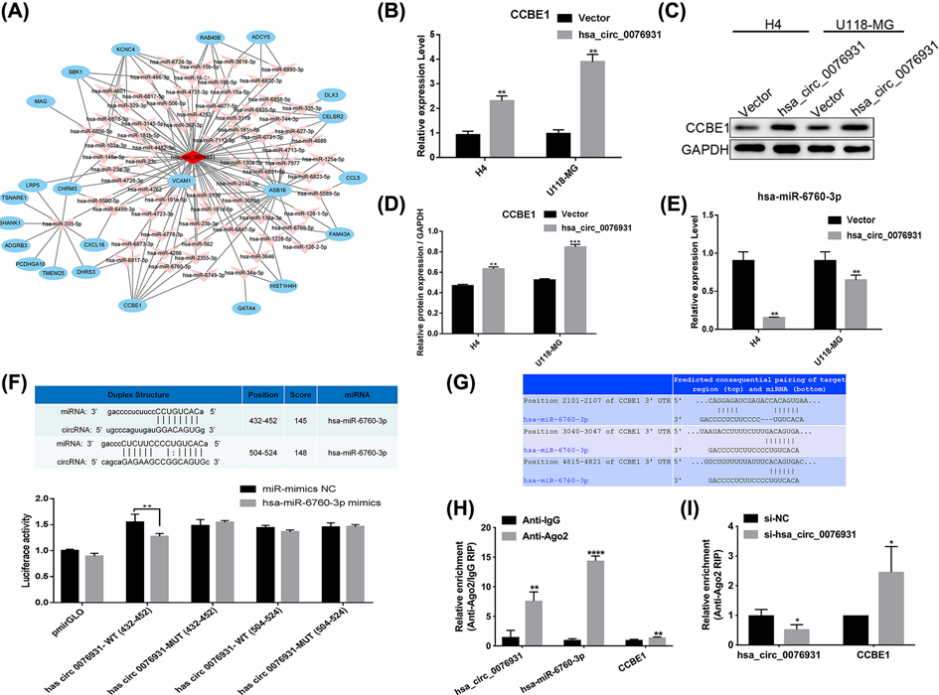
Hsa_circ_0076931/miR-6760-3p/CCBE1 axis is predicted to play important roles in the progression of glioma (**A**) circRNA/miRNA/mRNA ceRNA network analysis. (**B**) The level of CCBE1 in H4 and U118-MG cells was detected by qRT-PCR after transfection with hsa_circ_0076931. (**C**) The level of CCBE1 in H4 and U118-MG cells was detected by Western blot after transfection with hsa_circ_0076931. (**D**) Quantification of CCBE1 protein is shown. (**E**) The level of miR-6067-3p in H4 and U118-MG cells was detected by qRT-PCR after transfection with hsa_circ_0076931. (**F**) Schematic of the binding site between hsa_circ_0076931 and miR-6067-3p, and the luciferase activity of WT hsa_circ_0076931 3′ UTR or mutant hsa_circ_0076931 3′ UTR after transfection with miR-6067-3p mimics in 293T cells. (**G**) Schematic of the binding site between miR-6067-3p and CCBE1. (**H**) AGO2-RIP assay was used to analyze the expression of hsa_circ_0076931, miR-6760-3p, and CCBE1 in U118-MG cells. (**I**) After interference with hsa_circ_0076931, the expression of CCBE1 was tested by AGO2-RIP assay in U118-MG cells; *P<0.05, **P<0.01, ***P<0.001.

CircRNAs act as ceRNAs by sequestering target miRNAs and thereby diminishing the repressive effects on downstream molecules of miRNAs [23,24]. The ceRNA network analysis showed internal relationships between hsa_circ_0076931, 23 mRNAs, and predicted target miRNAs (Figure 6A). CCBE1 was predicted as a target gene of hsa_circ_0076931. The ceRNA network suggests that CCBE1 has many binding sites, consistent with literature reports showing that CCBE1 is down-regulated in tumors. We also confirmed by qRT-PCR and immunoblotting that elevated hsa_circ_0076931 induced a high level of CCBE1 in both U118-MG and H4 cells compared with controls (Figure 6B–D, Supplementary Figures S1 and 2). In addition, the qRT-PCR results show that elevated hsa_circ_0076931 decreased the level of miR-6760-3p (Figure 6E). Furthermore, we detected miR-6760-3p, which is predicted to have two potential binding sites to hsa_circ_0076931 by miRanda. When luciferase reporter assays were applied to determine whether miR-6760-3p directly targets hsa_circ_0076931, a remarkable reduction in luciferase reporter activity was detected in 293T cells co-transfected with miR-6760-3p mimics and hsa_circ_0076931 WT (432-452 bp) but not with the hsa_circ_0076931 WT (504-524 bp), hsa_circ_0076931 MUT (432-452 bp), or has_circ_0076931 MUT (504-524 bp) (Figure 6F). The potential binding sites of miR-6760-3p and CCBE1 were also shown (Figure 6G). Moreover, we used the AGO2-RIP assay to test whether hsa_circ_0076931, miR-6760-3p, and CCBE1 exist in the same RISC complex and precipitate endogenous AGO2 protein in cells. The results showed that the expression levels of hsa_circ_0076931, miR-6760-3p, and CCBE1 were higher in the AGO2 immunoprecipitation complex than those in the IgG immunoprecipitation complex, suggesting that hsa_circ_0076931, miR-6760-3p, and CCBE1 have certain regulatory effects in an AGO2-dependent manner (Figure 6H). We also found that compared with the si-NC group, hsa_circ_0076931 was down-regulated, whereas CCBE1 was up-regulated in the AGO2 immunoprecipitation complex from hsa_circ_0076931-silenced cells (Figure 6I). These experiments indicate that hsa_circ_0076931 might function as a sponge for miR-6760-3p to regulate CCBE1 in glioma progression.

### The expression of miR-6760-3p and CCBE1 in hsa_circ_0076931-overexpressed tumors of BALB/C nude mice and glioma tissues and relevance to prognosis

**Figure 7:**
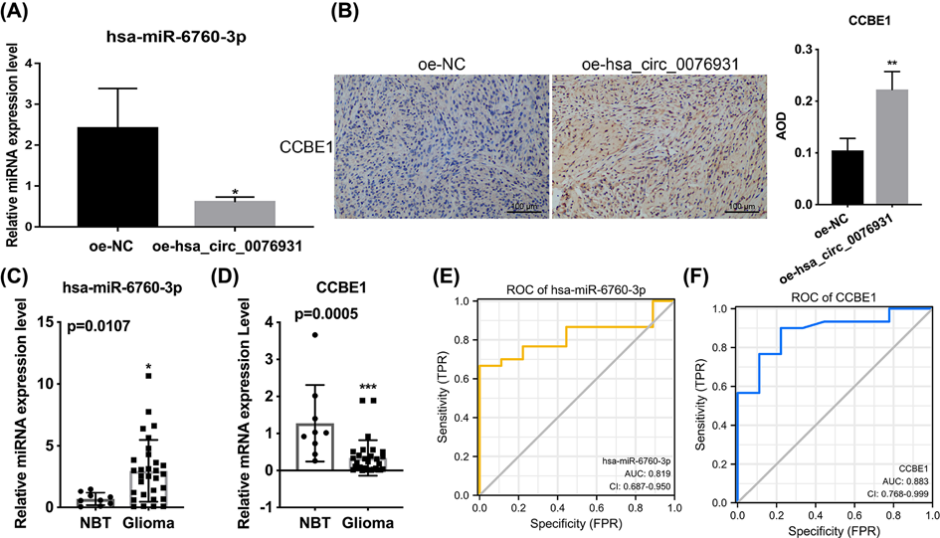
The expression of miR-6760-3p and CCBE1 in hsa_ circ_0076931-overexpressed tumors of BALB/C nude mice and glioma tissues and relevance to prognosis (**A**) The abundance of miR-6760-3p was quantified by qRT-PCR in circ_0076931-overexpressed mouse tumors. (**B**) The protein level of CCBE1 was assessed by immunoblot in circ_0076931-overexpressed mouse tumors. The RNA levels of miR-6760-3p (**C**) and CCBE1 (**D**) were examined by qRT-PCR in glioma and normal brain tissues. (**E** and **F**) The ROC curves of miR-6760-3p (AUC = 0.819) and CCBE1 (AUC = 0.883) in glioma patients; *P<0.05, **P<0.01, ***P<0.001.

Finally, we verified the expression of miR-6760-3p and CCBE1 *in vivo* and their relationship with the prognosis of patients with glioma. We found that miR-6760-3p was memorably reduced in the hsa_circ_0076931 overexpression group versus the NC group (Figure 7A). IHC showed that CCBE1 was memorably up-regulated in mouse tumors with hsa_circ_0076931 overexpression (Figure 7B). In addition, compared with NBT, miR-6760-3p was notably up-regulated, whereas CCBE1 was dramatically down-regulated in glioma tissues as measured by qRT-PCR (Figure 7C,D). Meanwhile, we found that the AUC of miR-6760-3p was 0.819 (Figure 7E), and the AUC of CCBE1 was 0.883 (Figure 7F) in glioma patients. Moreover, we discovered that there was no difference in the expression of hsa_circ_0076931, miR-6760-3p, and CCBE1 among astrocytoma <= II, Astrocytoma >= II, and glioblastoma IV tissues (Supplementary Figure S3). Thus, we suggest that hsa_circ_0076931 overexpression can down-regulate miR-6760-3p and up-regulate CCBE1 *in vivo*, and miR-6760-3p and CCBE1 have relevance to the prognosis of glioma patients.

## Discussion

Emerging evidence suggests that dysregulation of circRNA profiles is closely related to pathological conditions, such as neurological disorders, diabetes, cardiovascular diseases, and cancer [3,25]. In the present study, we are the first to reveal the circRNA profile in gliomas by employing comparative RNA sequencing of gliomas and NBT. A total of 507 differentially expressed circRNAs were identified between glioma and NBT samples. Consistent with previous studies of circRNA profiling [26–28], most circRNAs (88.59%) were derived from protein-coding exon circularization, indicating that various additional transcripts can be produced from precursor mRNAs. In addition, most differentially expressed circRNAs decreased in glioma compared with the NBT samples. Four circRNAs (hsa_circ_0007694, 0008016, 0076931, and 0076948) were selected for validation using qRT-PCR in six pairs of glioma and NBT tissues. The results showed that hsa_circ_0007694 increased whereas hsa_circ_0076931 decreased prominently in glioma tissues compared with NBT. Based on these data, hsa_circ_0076931 was chosen for follow-on analysis. Furthermore, we identified the major notably changed GO terms, most correlated KEGG pathways, and predicted circRNA/miRNA/mRNA interactions. The potential roles of the aberrantly expressed circRNAs in the progression of glioma make them appealing to use as novel biomarkers or effective therapeutic targets.

One of the abundant circRNAs we identified, termed hsa_circ_0076931, is derived from exons 4-8 of the BAI3 gene (NM_001704) and has a junction of 768 nt. The abundance of hsa_circ_0076931 in glioma samples was lower than in the NBT samples. In addition, hsa_circ_0076931 expression was negatively correlated with histological grade. Additionally, we found that elevated hsa_circ_0076931 inhibited proliferation, migration, and invasion while promoting apoptosis of glioma cells *in vitro*. To further investigate the tumor-suppressing effects of hsa_circ_0076931 *in vivo*, hsa_circ_0076931-overexpressed U118-MG and wild-type U118-MG cells were injected into nude mice by hypodermic injection. Strikingly, hsa_circ_0076931 overexpression dramatically reduced mean tumor volume and weight. Ki67 is a nuclear protein used as a cell proliferation marker in human tumors [29]. We found that hsa_circ_0076931 dramatically decreased the percentage of Ki67-positive cells. These results indicated that hsa_circ_0076931 is a promising therapeutic target for glioma. However, the expression and role of hsa_circ_0076931 in other diseases remain unclear and should be urgently identified.

CircRNAs are thought to act as miRNA sponges that diminish the repressive effects of miRNAs on downstream molecules [30–32]. MiRNAs are single stranded RNAs composed of 20-25 nt that can change post-transcriptional gene expression [33,34]. Our studies identified miR-6760-3p as a potential target of hsa_circ_0076931 and showed that hsa_circ_0076931 suppressed the level of miR-6760-3p. In addition, the binding site between hsa_circ_0076931 and miR-6760-3p was confirmed by luciferase reporter assays. Furthermore, CCBE1 was predicted to be a target of miR-6760-3p. Until now, studies on CCBE1 have mainly focused on the lymphatic system. CCBE1 plays a vital role in the development of the vascular system in the embryo and early childhood [35]. Mutation of the CCBE1 gene causes lymphedema, abnormal development of lymphatic vessels, and other related lymphoid diseases [36–38]. CCBE1 promotes VEGF-C transformation from precursors to active forms, combining with VEGFR-3 to execute biological roles [39,40]. The only study on the role of CCBE1 in cancer shows that CCBE1 acts as a tumor suppressor gene in ovarian cancer due to methylation causing gene expression silencing [40]. Therefore, the specific mechanism of CCBE1 in the progression of glioma tumors remains unclear. Our study showed that elevated hsa_circ_0076931 induced a high level of CCBE1. Thus, we speculate that hsa_circ_0076931 might function as a sponge for miR-6760-3p to regulate CCBE1 in glioma progression. Nevertheless, the present study has some limitations. First, because the sample size of the present study was relatively small, it lacked the statistical power for multiple regression analysis. Second, the exact regulation between hsa_circ_0076931, miR-6760-3p, and CCBE1 lacks experimental validation, such as pull-down, chromatin immunoprecipitation. Third, the impact of the putative hsa_circ_0076931/miR-6760-3p/CCBE1 axis on glioma progression should be confirmed by rescue experiments.

## Conclusion

In summary, we presented differentially expressed circRNAs between glioma and NBT. The level of hsa_circ_0076931 markedly decreased in glioma samples compared with NBT samples. Furthermore, elevated hsa_circ_0076931 inhibited proliferation, migration, and invasion of glioma cells *in vitro*. Additionally, we showed that hsa_circ_0076931 can down-regulate miR-6760-3p through direct binding and can upregulate CCBE1. Therefore, miR-6760-3p and CCBE1 might be the regulatory mechanism of hsa_circ_0076931 in glioma.

## Informed Consent

Written informed consent was obtained from the guardians of all subjects.

## Supplementary Material

Supplementary Figures S1-S3

## Data Availability

The sequencing data were deposited in BioProject (ID: PRJNA746438) at https://www.ncbi.nlm.nih.gov/bioproject/?term=prjna746438.

## Abbreviations

ceRNA: competitive endogenous RNA
circRNA: circular RNA
IHC: immunohistochemistry
NBT: normal brain tissues
