# Supplementary material for: Hsa_circ_0076931 suppresses malignant biological properties, down-regulates miR-6760-3p through direct binding, and up-regulates CCBE1 in glioma
Source: Biosci Rep. 2022 Jan 6;42(1):BSR20211895. doi: 10.1042/BSR20211895 (PMC8738865; doi:10.1042/BSR20211895)
Supplement: Supplementary Figures S1-S3 [file BSR-2021-1895_supp.pdf]

Supplementary Materials

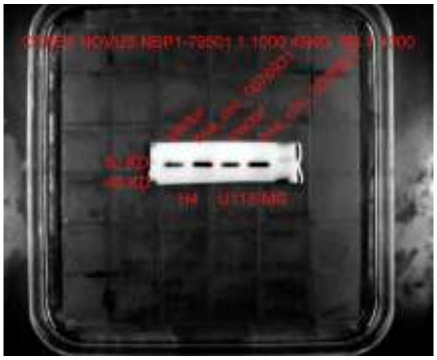

Supplementary Figure 1. The original immunoblots of CCBE1.

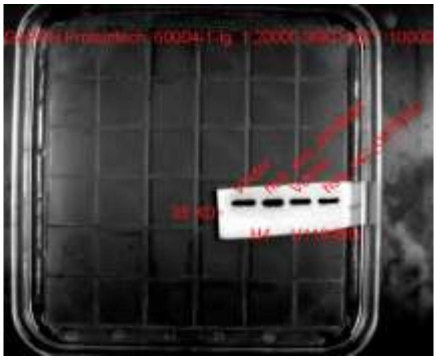

Supplementary Figure 2. The original immunoblots of GAPDH.

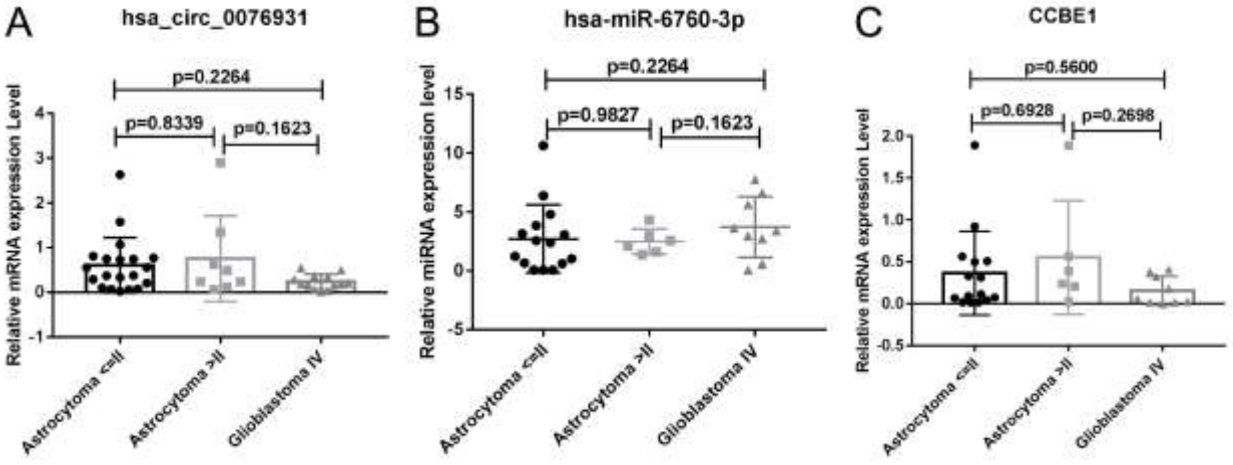

Supplementary Figure 3. The relationship between WHO grade and hsa_circ_0076931, miR-6760-3p, or CCBE1 in glioma. (A) hsa_circ_0076931, (B) miR-6760-3p, and (C) CCBE1 expression were analyzed by qRT-PCR in different grades of gliomas (Astrocytoma <=II; Astrocytoma >II; Glioblastoma IV).
